# Relationship of peer specialists to mental health outcomes in South Florida

**DOI:** 10.1186/s13033-018-0239-6

**Published:** 2018-10-20

**Authors:** Daniel Castellanos, Mayte Capo, Diana Valderrama, Melissa Jean-Francois, Aniuska Luna

**Affiliations:** 1 Department of Psychiatry & Behavioral Health, Herbert Wertheim College of Medicine, Florida International University, 11200 SW 8th St, AHC1 349, Miami, FL 33199 USA; 2 South Florida Behavioral Health Network, 7205 Corporate Drive, Suite 200, Miami, FL 33126 USA; 3 Robert Stempel College of Public Health & Social Work, Florida International University, 11200 SW 8th St, AHC1 349, Miami, FL 33199 USA

**Keywords:** Peer specialists, Mental health, Outcomes

## Abstract

**Background:**

In recent years the use of peer specialists in the delivery of mental health of care across the US has increased. Although data on the benefits of using peer specialists is limited and/or equivocal, states are making policy and funding decisions to support the expansion of peer specialist services. This data is even more limited in the state of Florida where no studies were found to document the effect of peer specialists on mental health care outcomes. The purpose of this study was to assess whether local decisions to use peer specialists can be supported through the measurement of outcomes of service utilization and mental health functioning when peer specialists are involved in the treatment of individuals living with serious mental illness.

**Methods:**

The study was conducted using service data collected by South Florida Behavioral Health Network (SFBHN). SFBHN is the Managing Entity for publicly funded mental health and substance abuse services in Miami-Dade and Monroe Counties in Florida. We compared mental health outcomes and service utilization between individuals who received peer specialist services (n = 367) and a treatment as usual group (n = 1468) matched on gender, age and severity of diagnosis in the period July 2013 and June 2015. Multilevel models were used to evaluate the functioning outcomes between the groups. Service utilization was assessed using negative binomial regression.

**Results:**

Individuals in the treatment group receiving peer specialist services utilized more ambulatory/lower levels of care services and had more frequent crisis stabilization unit admissions. Those in the treatment group also displayed more functional difficulties with a variety of practical activities, employment and housing and violent temper, hostility, threatening behaviors.

**Conclusions:**

The findings of the study further support existing evidence documenting the mixed benefits of using peer services compared to treatment as usual care. Policy makers and other stakeholders are encouraged to advance mental health recovery by examining outcomes more comprehensively. Future research should include examination of the subjective benefits of peer support for recipients, understanding the impact on service utilization and a better definition of the roles, supervision and expectations of peer support programs.

## Background

Over the past several years utilization of peer support has become a common practice and an important component in the recovery of people living with serious mental illness (SMI), with many services and agencies being completely consumer run and governed [1, 2]. Consumers have played an increasingly significant role by actively participating in the planning, evaluation, research, training and delivery of mental health services throughout the world [3, 4]. Since 2003, this increase can be attributable in part to the recommendation of the President’s New Freedom Commission on Mental Health for the integration of peer support services into the continuum of community care [5]. These programs and services have become so popular that waitlists have been implemented at many locations [6]. Therefore, States are making policy and funding decisions to support the expansion of peer specialist services.

Research studies examining peer specialists, however, have not kept pace with its broad proliferation though some localized reports have been produced [7]. The benefits that result from peer support have been sparsely documented yet the integration of peer specialists as paid positions in treatment continues to be encouraged [8]. Research findings on the relationship between peer support services (non-certified peer specialists and other types of peer support) and mental health outcomes also remain highly variable [2, 9]. Furthermore, funders are now increasingly demanding evidence that program models work.

The authors of the current article, therefore, decided to investigate and assess if the intervention of peer specialists indeed merits their widespread use. Assessment was based on the measurement of outcomes of service utilization and mental health functioning when peer specialists are involved in the treatment of individuals living with serious mental illness. The findings pinpoint to a need for regulation, standardization, and assessment of education, certification, and supervision of peer specialists and the programs they are involved with.

Studies have found a significant relationship between peer support and certain outcomes such as recovery, increased health self-management, decreased depression symptoms, and increased knowledge, motivation and hope [6, 10]. Yet most of these outcome measures relied on a consumer’s self-reports or an individual’s perception of an outcome.

The majority of studies report consumers having no additional benefits from peer support services when compared to usual care. A meta-analysis of 18 trials with varying methodologies and program content including 5597 participants found little or no evidence that peer support was associated with positive effects on hospitalization, overall symptom improvement or satisfaction with services [11]. The investigators involved in the analysis did find some evidence that peer support was associated with positive effects on measures of hope, recovery and empowerment at and beyond the end of the intervention, although this was not consistent within or across different types of peer support [11]. Other studies have compared outcomes for individuals who received usual treatment and those with peer intervention. These studies reported no difference in quality of life, self-efficacy, social support, functioning, disability and burden of care for persons who received the peer interventions [1, 12, 13].

The relationship between peer support and consumer service utilization has been explored to a lesser extent. The majority of the evidence on admission rates reveals mixed or positive results. Clarke and colleagues [14] found no impact from peer support services in retention rates, frequency of face-to-face contact with peers, crisis episodes, hospital episodes, hospital bed days, and risk of hospitalization. They did report a significant relationship between consumer case management and increased time to first psychiatric hospitalization and decreased number of consumers visiting the emergency department (ED). Chinman et al. [15], Min et al. [16], Forchuk et al. [17], and Lawn et al. [18] found similar support for the benefit of peer specialist intervention and rehospitalization rates. These authors found that rehospitalization was reduced with the involvement of peer specialists in care.

Other studies that addressed service utilization found no significant impact from peer support services on this type of outcome [1, 19–21]. For instance, Landers and Zhou [22] examined the association between peer support and the costs of psychiatric hospitalization, crisis stabilization, and total Medicaid costs in Georgia. They found that peer support was associated with higher total Medicaid cost, higher Medicaid drug cost, higher Medicaid professional cost, and lower facility cost. Cabassa et al. [23], in their review of the literature on the subject, further illustrated the dearth of studies adequately showing the impact of peer specialist intervention on health outcomes.

In spite of the inconsistency in the findings, as stated previously, the use of peer specialists continues to expand. In the state of Florida peer specialists are part of the network of mental health care workers described by the Department of Children and Families [24]. Yet no study was found reporting on the benefits of peer specialists throughout the state.

The Broward Behavioral Health Coalition [7], for example, documented that the State of Florida reports 555 certified peer specialists and an unknown number of non-certified peer specialists in the workforce. The report identified challenges with high turnover rates, wide variations in salary ($10–$26/h) and a lack of standardized training for peer specialist supervisors. Other difficulties identified around the peer specialist workforce include how a criminal background is a major barrier to peer certification, having peer specialists maintain consistent self-care, boundaries and stage of change without jeopardizing their recovery, a need for more frequent one-on-one supervision due to limited work experience and finding qualified peers who can pass a substance abuse screening [7]. Similarly, the Florida Peer Services Handbook [24] provides a better understanding of the role of peer specialists and explores strategies for recruiting, hiring and supervising peer specialists.

The findings and descriptions of programs in these localized reports attest to challenges in the professionalization, implementation of quality and outcome initiatives, and expansion of utilization of peer specialists, *not* to their effectiveness in treatment approaches. As the utilization of peer specialists spreads then a study such as the one reported on here represents a necessity to assess and evaluate the rehabilitation approach involving the use of peer services; that may affect the cost and long term impact of mental health care.

## Methods

### Definition of peer specialist

“Peer support providers”, “peer workers”, “peer providers”, “peer support specialists”, and “recovery coaches” however, are terms frequently used interchangeably with that of “peer specialists”. The United States (US) Substance Abuse and Mental Health Services Administration (SAMHSA) [25] and the State of Florida officially utilize the term “peer specialist” used throughout this study.

### Data source

The study was conducted using service data collected by South Florida Behavioral Health Network, the state of Florida’s Managing Entity charged with the oversight of over 80 million dollars in publicly-funded mental health and substance abuse services in Miami-Dade and Monroe Counties, Florida. SFBHN requires all subcontracted service providers to provide monthly data for all of the consumers served through the network. Service providers upload their data through a secure remote connection into the SFBHN network drive.

### Sample

Eligibility criteria for individuals to receive SFBHN publicly funded services include: (1) being diagnosed with a serious mental illness with one of the following International Classification of Diseases (ICD-9-CM) diagnosis: Schizophrenic Disorders, Mood Disorders, Delusional Disorders, Other Non-Organic Psychoses/Psychotic Disorders; or (2) meeting criteria for involuntary evaluation and/or hospitalization as defined under state law; and (3) the individual’s income needs to be at or below the 150% poverty line. SFBHN began providing reimbursement for peer specialist services in July, 2013. Individuals 18 years and older who received services within the network between July 2013 and June 2015, and did not receive any peer specialist services previous to the study period were eligible.

A total of 24,933 subjects were potentially identified for inclusion in the study. The group of individuals receiving peer specialist services was considered the treatment (Tx) group and those not receiving peer specialist services were included in the treatment as usual (TAU) group. We identified 374 (1.5%) individuals as having received peer specialist services.

To estimate the sample size needed for this study, power was set at 80%, the significance level (α) was set at 0.05, and three standardized effects were explored to estimate the sample size. Individuals in the sample were matched with regard to age, gender, race and ethnicity. Persons classified with multiple races and ethnic groups were excluded from the sample due to challenges in identifying their race and ethnicity accurately. Consequently, seven persons in the treatment group and 1232 in the TAU group were eliminated from the study, reducing the treatment group total to 367 and the unmatched TAU total to 23,327. A Chi Square test was run to ensure the two groups’ demographic profiles were not significantly different.

The data was matched using a 1–4 ratio (1 case to 4 controls). The 1–4 ratio was used because little statistical power is gained by increasing the ratio of controls to cases [26]. The final sample size totaled 1835 (n = 367 for the treatment and n = 1468 for TAU group).

SFBHN serves an area in Florida with a predominance of diverse Hispanic, ethnic, and racial minority groups. Although these groups are underrepresented in previous studies addressing the use of peer specialists in the treatment of people with serious mental illness [23], the design and focus of our study did not permit examination of linguistic and cultural factors. This information as collected (Table 1) for our study was for descriptive rather than comparative analysis purposes; its relevance in relation to peer specialist effectiveness was thus not considered in assessment.

**Table 1:** Demographic profile for treatment and treatment as usual groups

Variables	Treatment	TAU	N	%
	n = 367	n = 1468		
	n	%		
Age				
18–24	40	11	160	11
25–44	1134	37	537	37
45–64	178	48	712	48.5
65+	15	4	59	4
Race				
Asian	2	0.5	7	0.5
Black	114	31	455	31
Multi-racial	23	6	88	6
White	228	62	910	62
Am Indian/Alaska	0	0	0	0
Hawaiian/Pacific	0	0	0	0
N/A	0	0	0	0
Gender				
Female	143	39	572	39
Male	224	61	896	61
Ethnicity				
Cuban	98	27	396	27
Haitian	5	1	14	1
Hispanic	96	26	382	26
Mexican	1	0.3	4	0.3
Mexican–American	0	0	0	0
Puerto Rican	7	2	29	2
None of the above	1160	44	646	44

To minimize the possibility of confounding, individuals in the treatment group were matched based on gender, age and diagnosis with the TAU group. Performing a frequency matching procedure to create a TAU group that is similar to the treatment group, in age and severity of diagnosis decreases the likelihood of detecting an erroneous relationship between receiving peer specialist services and mental health outcomes that could be a result of chronicity. Using ICD-9-CM definitions, subjects were grouped into nine general diagnostic categories which consisted of Psychosis/Schizophrenia, Bipolar Disorder, Depression, Anxiety, Post-traumatic Stress Disorder (PTSD), Adjustment Disorders, Other or unspecified affective psychosis, unspecified mental disorder, and unknown or deferred (Table 2).

**Table 2:** Diagnosis of treatment and treatment as usual groups

Variable	Treatment	TAU	%
	(n = 367)	(n = 1468)	
	N	N	
Diagnosis			
Psychosis/schizophrenia	171	685	47
Bipolar disorder	55	220	15
Depression	114	455	31
Anxiety	11	44	3
PTSD	4	16	1
Adjustment disorder	9	36	2
Unspecified mental disorder	3	12	0.8

### Measures

Service utilization and measures of functioning was extracted from the information submitted by SFBHN providers.

### Service utilization data

Mental health services can be divided in two types: (1) high level of care (inpatient), including admission to a crisis stabilization unit (CSU) and/or a short term residential treatment (SRT) program and (2) low level of care (outpatient) services that include individual/group therapy, aftercare (group/follow up), in-home and on-site services, intervention individual/group, clubhouse services, supported employment, supported housing, mental health comprehensive (individual/group), and medication management. Higher level of care services are considered to be more intensive and costly when compared to lower level of care services. Utilization of inpatient services was measured using number of admissions and length of stay, and outpatient services were measured using the number of encounters (frequency of services).

### Functioning data

Participants’ Functional Assessment Rating Scale (FARS) scores that were available within the study period were extracted from the database. The FARS is a standardized clinical evaluation tool that assesses individuals’ cognitive, social and role functioning [27]. The FARS generates severity ratings for 16 different functional domains: depression, hyper affect, cognitive performance, traumatic stress, interpersonal relationships, family environment, work/school, anxiety, thought process, medical/physical, substance use, family relationships, socio-legal, activities of daily living (ADLs) functioning, danger to self, and security/management needs. The scale is accepted as a validated measure of mental health care outcomes often combined with the documentation by providers of demographic data. Providers are required to administer the FARS at the consumers’ admission into a treatment episode, every 6 months thereafter and at discharge.

The Florida Department of Children and Families (DCF) requires the collection of individual level data on employment status of each person who is the recipient of any service in a mental health program funded by DCF. Data are collected in the DCF Mental Health Outcome Form at admission into the treatment episode, every 3 months thereafter and at discharge. Residential and employment status obtained from the data reported in this statewide tool are used as further measures of functioning.

### Data analyses

Using SPSS 18.0 statistical software, multilevel models were used to detect differences in FARS scores during the study period between subjects who received peer specialist services (treatment group) and subjects who did not receive peer specialist services (TAU group). Several multilevel models were fitted to predict the FARS scores outcome at the end of the study. Likelihood ratio tests were used to compare and identify the model with the best fit.

In all multilevel models examined time was treated as a random variable to take into account the varying duration of the episodes of care among the participants. An episode of care was defined as active treatment utilization until the consumer was discharged from care or 70 days lapsed without receiving services. A multilevel model was also used to investigate the differences in length of stay in a CSU as well as the differences in employment and residential status between the two groups.

Service utilization data was utilized to estimate a negative binomial model. A negative binomial model is derived from a Poisson distribution and is used to model count data. A negative binomial regression is robust to issues of over dispersion observed in the service utilization data.

### Ethics

The Health Sciences Institutional Review Board of Florida International University approved this study for the use of human subjects via the Expedited Review process (Protocol Approval #: IRB-15-0453).

## Results

### Service utilization outcomes

Individuals in the treatment group were more likely to utilize low level of care services than individuals in the TAU group. Individuals in the treatment group were significantly more likely to use therapy (IRR = 3.1), case management (IRR = 3.7), in-home and on-site services (IRR = 5.1) and intervention group/individual (IRR = 4.4) when compared to the TAU group. Individuals in the treatment group were less likely to use Comprehensive Community Service Teams (IRR = 0.098) and medication management (IRR = 0.636). They were also less likely to use aftercare services (IRR = 0.900), however, this was not statistically significant (Table 3).

**Table 3:** Outpatient frequency of services—treatment vs treatment as usual groups

Services	*B*	*SE*	*IRR*	95% *CI*	*p*
Therapy services (group and individual)	1.134	0.17	3.108	2.228, 4.337	< 0.001*
Case management services	1.309	0.168	3.703	2.664, 5.149	< 0.001*
Aftercare	− 0.105	0.229	0.9	0.574, 1.411	0.646
In-home and on-site service	1.631	0.367	5.109	2.487, 10.496	< 0.001*
Intervention (group and individual)	1.476	0.148	4.377	3.277, 5.847	< 0.001*
Comprehensive community service teams	− 2.327	0.52	0.098	0.035, 0.270	< 0.001*
Med management	− 0.453	0.101	0.636	0.521, 0.775	< 0.001*

* p < 0.05

**Fig. 1:**
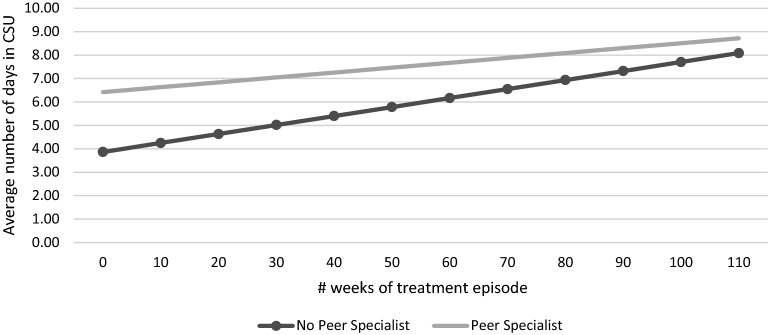
Length of crisis stabilization unit (CSU) stay

Individuals in the treatment group were also more likely to be admitted to high level of care services, CSU (IRR = 1.3) and short term residential/SRT (IRR = 1.3), although this prediction was not statistically significant for SRT (Table 4). Individuals in the treatment group had an average length of CSU stay that was almost twice as that of the TAU group at baseline (Table 5). As the time of treatment progressed, the average length of stay increased at a lower rate for individuals in the treatment group when compared with the TAU group. The latter interaction, however, was not statistically significant (see Fig. 1).

**Table 4:** Inpatient frequency of admissions—treatment vs treatment as usual groups

Services (treatment) (comparison as reference)	*b*	*SE*	*IRR*	95% *CI*	*p*
Crisis stabilization unit admissions (short term)	0.246	0.094	1.280	1.064, 1.539	0.009*
Long term residential admissions (long term)	0.247	0.185	1.280	0.890, 1.841	0.182

* p < 0.05

**Table 5:** CSU average length of stay—treatment vs treatment as usual groups

	*b*	*SE*	95% *CI*	*p*
Intercept	3.863	0.303	3.267, 4.459	< 0.001*
Time	0.038	0.023	− 0.007, 0.084	0.095
Treatment group	2.558	0.708	1.167, 3.949	< 0.001*
Time*treatment group	− 0.018	0.045	− 0.108, 0.073	0.699

* p < 0.05

### Functioning outcomes

At baseline, individuals in the treatment group were functioning better than individuals in the TAU group in the domains of anxiety, traumatic stress, and interpersonal relationships. Meanwhile, subjects in the TAU group were functioning better than individuals in the treatment group in the domains of ADL functioning, ability to care self, and security/management needs. For all other categories the groups did not differ at baseline. A FARS score that decreases indicates that the individual is functioning better in the specific functioning domain. Over time, individuals in the treatment group showed improvement (FARS score decreased) in the domain of Interpersonal Relationships (− 0.0002, p = 0.01) when compared to the TAU. Nonetheless, over time, individuals in the treatment group displayed significantly worsening in the majority of FARS domains when compared to the TAU group. Functioning worsened for the treatment group in the domains of depression, hyper affect, cognitive performance, traumatic stress, interpersonal relationships, family environment, work/school, anxiety, thought process, medical/physical, substance use, family relationships, socio-legal functioning, and danger to self. Individuals in the treatment group also displayed worsening in the domains of ADL functioning, danger to others and security/management needs; however, these changes were not statistically significant (Table 6).

**Table 6:** Changes of FARS functioning domains throughout treatment–treatment vs treatment as usual group

	*b*	*SE*	95% *CI*	*p*
Depression	0.009	0.003	0.003, 0.015	0.004*
Anxiety	0.013	0.003	0.007, 0.020	< 0.001*
Hyper affect	0.006	0.003	0.001, 0.012	0.014*
Thought process	0.009	0.003	0.003, 0.014	0.001*
Cognitive performance	0.006	0.003	0.001, 0.011	0.018*
Medical/physical	0.006	0.003	0.001, 0.012	0.024*
Traumatic stress	0.007	0.003	0.002, 0.013	0.007*
Substance use	0.007	0.002	0.002, 0.011	0.003*
Interpersonal relationships	0.025	0.007	0.011, 0.038	< 0.001*
Family relationships	0.012	0.003	0.007, 0.018	< 0.001*
Family environment	0.011	0.003	0.005, 0.017	< 0.001*
Socio-legal	0.011	0.003	0.005, 0.017	< 0.001*
Work/school	0.022	0.003	0.016, 0.028	< 0.001*
ADL functioning	0.002	0.002	− 0.002, 0.006	0.401
Ability to care for self	0.006	0.002	0.002, 0.010	0.008*
Danger to self	0.002	0.002	− 0.001, 0.005	0.272
Danger to others	0.001	0.001	0.00, 0.00	0.415
Security/management needs	0.001	0.002	− 0.003, 0.005	0.508

*FARS* Functional Assessment Rating Scale

* p < 0.05

Peer specialists’ intervention did not seem to affect the likelihood of being employed (OR = 0.983, p = 0.009) when compared to individuals in the TAU group. In addition, individuals in the treatment group did not display significant differences in living dependently (OR = 1.010, p < 0.001) as treatment progressed. Homelessness status reflected a similar pattern (OR = 1.006, p = 0.045).

## Discussion

Our study is the first to examine the relationship between peer specialists and mental health outcomes by assessing service utilization and functioning outcomes in a large cohort of individuals. The findings show that individuals receiving peer specialist services utilized more ambulatory/lower level of care services, but were less likely to use Comprehensive Community Service Teams and medication management services. The group receiving peer specialist services was admitted to crisis stabilization units more frequently. At baseline, those receiving peer specialist services had an average length of CSU stay that was almost twice as that of the TAU group. Subjects in both groups exhibited an increase in the length of CSU admission days throughout their treatment episodes, with those in the TAU group increasing at a higher but not significant rate (Fig. 1).

The presence of peer specialist services was associated over time with significant worsening in the majority of functioning domains when compared to the TAU group. On the other hand, our data did not reveal differences between the groups in the likelihood of living dependently, becoming homeless or being employed. Our study differed from others by utilizing a standardized instrument (FARS) to measure functioning rather than self-report as the primary measure of functioning [28, 29].

Using data from real life practice settings, our study adds to the evidence base indicating that there were unclear benefits from peer services compared with treatment as usual care. The peer specialist group utilized more ambulatory/lower level services, utilized crisis stabilization services more frequently and displayed an overall worsening in functioning. Engagement of more ambulatory services would be an expected outcome of provision of peer specialist services. A corresponding decrease in the frequency of acute care admissions and improvement in functioning would have been desired consequences.

It seems likely that peer services may be beneficial to some individuals in certain circumstances and settings, though what the nature of these circumstances is not discernible yet. In our study, the mere involvement of a peer specialist, with their varying roles and duties, in the treatment plan of an individual with a serious mental illness, appears to be a less than optimal measure of the impact or relationship to mental health outcomes.

Outcomes for individuals experiencing serious mental illness can be inherently personal and recovery can be complex. Each person who received peer specialist services lived their own clinical and service delivery experience. Encouragement, confidence and/or satisfaction could have resulted in the use of more outpatient services. In addition, peers can help improve access, adherence and/or utilization by facilitating the navigation of the complexities of the behavioral health system.

The decision to select which individuals received peer specialist services was determined by each particular service agency. We controlled for chronicity by matching diagnosis between the two groups but could not determine qualitative differences between individuals within each diagnostic group. Some of the variability of our results (e.g., the peer specialist group using crisis interventions services more frequently) could be explained by the different complexities and variations in the clinical presentations of individuals within each diagnostic group.

All individuals in our study population had incomes 150% below the US. Federal poverty level. The relationship between serious mental illness and poverty is complicated. Poverty may intensify the experience of mental illness or may also increase the likelihood of the onset of mental illness. At the same time, experiencing mental illness may also increase the chances of living below the poverty line [30]. Poverty and other social determinants of health are important, complex factors in addressing health, which are not fully understood. The examination of the effects of these factors went beyond the scope of this investigation.

The arbitrariness of the findings, here and in other studies, may also be attributed to a mirrored diversity and inconsistency in training and intervention of peer specialists in the state of Florida and throughout the United States [7, 23, 29, 31–33]. The effect of a lack of regulation and standardization in training of peer specialists can be further illustrated in descriptions of trainings across agencies in reports such as the Peer Support Workforce Report generated by the Broward Behavioral Health Coalition [7]. As educators, mental health practitioners and providers, and as researchers the authors of this study consider problematic the potential pitfalls hinted at the findings in relation to the lack of regulation and standardization in training. For peer specialists to be considered as moderators of improved mental health for persons living with a serious mentally illness, consistent and clearer roles, functions and expectations would be required. Peer specialists are a “helping”, not a clinical profession. As such, they do not have the same kinds of standards that a licensed clinician (e.g. mental health counselor, social worker) would have. The link and significance between peer specialists’ training, role description and practices and the outcome and effectiveness of their intervention in the treatment of patients with serious mental illnesses should be consequently investigated in more depth. The inconsistent results of this research study highlight the need for better data to clarify which benefits exist in this area.

## Limitations

A number of issues can exist with the collection of the “real-world” data utilized in our study. Oversight of the quality of data, incomplete databases, more chances of bias and confounding can exist. For example, all of the information was collected in English. The majority of our study population was Hispanic and it is not known how many individuals were bilingual or if clinicians had to translate questions for the required data collection. By state law all individuals in our study population had incomes 150% below the poverty level. Consequently, caution should be used when generalizing these results to individuals with higher incomes.

Peer specialists are not a profession certified, regulated or monitored by any governmental Florida agency to ensure their competency and safety in service to the people of the state [34]. The Florida Certification Board, a non-profit organization, provides “Recovery Peer Specialist Certifications”; is not, however, formally recognized by the State to assure its graduates’ fitness and competence in providing peer specialist services [35]. As a result, and based on the findings to the current study and the review of the literature, we believe that such a lack of regulation in practice, training and certification of peer specialists obstructs effective measurement and assessment of intervention outcomes for the population studied [23, 29, 33]. This also hinders the generalizability of the effectiveness of peer specialists’ involvement in treatment across facilities and programs others than the ones studied in this investigation. Our study examined the outcomes of service utilization and mental health functioning associated with the provision of peer specialist services. Future studies should consider examining outcomes more comprehensively by including subjective, recovery oriented measures (the “personal journey”) along with utilization and other mental health outcomes.

## Conclusions

To this point the benefits of peer services, such as the provision of mutual aid, encouragement of self-determination and personal responsibility and a focus on health and wellness have been documented [29, 36]. The importance of peer specialist services to objective consumer mental health outcomes, however, has not been well established. Policy makers and other stakeholders are encouraged to advance mental health recovery by examining outcomes more comprehensively. Future research should include examination of the subjective benefits of peer support for recipients, understanding the impact on service utilization and a better definition of the roles, supervision and expectations of peer support programs. In today’s changing healthcare environment, this information is vital to policymakers and program developers as they continue to build a financially sustainable infrastructure necessary to meet the needs of individuals living with a serious mental illness.

## Abbreviations

SMI: serious mental illness
SAMHSA: Substance Abuse and Mental Health Services Administration
SFBHN: South Florida Behavioral Health Network
ICD: International Classification of Diseases
PTSD: post-traumatic stress disorder
CSU: crisis stabilization unit
SRT: short term residential treatment
FARS: Functional Assessment Rating Scale
ADLs: activities of daily living
DCF: Department of Children and Families
TAU: treatment as usual
Tx: treatment group
